# GRIN2B Gene and Associated Brain Cortical White Matter Changes in Bipolar Disorder: A Preliminary Combined Platform Investigation

**DOI:** 10.1155/2013/635131

**Published:** 2013-12-30

**Authors:** Carissa Nadia Kuswanto, Min Yi Sum, Christopher Ren Zhi Thng, Yi Bin Zhang, Guo Liang Yang, Wieslaw Lucjan Nowinski, Yih Yian Sitoh, Chian Ming Low, Kang Sim

**Affiliations:** ^1^Research Division, Institute of Mental Health, 10 Buangkok View, Singapore 539747; ^2^NUS High School of Mathematics and Science, 10 Clementi Avenue 1, Singapore 129957; ^3^Department of Pharmacology, Yong Loo Lin School of Medicine, National University of Singapore, 1E Kent Ridge Road, Singapore 119228; ^4^Biomedical Imaging Laboratory, Singapore Bioimaging Consortium, Agency for Science, Technology and Research, 11 Biopolis Way, Singapore 138667; ^5^Department of Neuroradiology, National Neuroscience Institute, 11 Jalan Tan Tock Seng, Singapore 308433; ^6^Department of Anesthesia, Yong Loo Lin School of Medicine, National University of Singapore, 1E Kent Ridge Road, Singapore 119228; ^7^Department of General Psychiatry, Institute of Mental Health/Woodbridge Hospital, 10 Buangkok View, Singapore 539747

## Abstract

Abnormalities in glutamate signaling and glutamate toxicity are thought to be important in the pathophysiology of bipolar disorder (BD). Whilst previous studies have found brain white matter changes in BD, there is paucity of data about how glutamatergic genes affect brain white matter integrity in BD. Based on extant neuroimaging data, we hypothesized that GRIN2B risk allele is associated with reductions of brain white matter integrity in the frontal, parietal, temporal, and occipital regions and cingulate gyrus in BD. Fourteen patients with BD and 22 healthy controls matched in terms of age, gender and handedness were genotyped using blood samples and underwent diffusion tensor imaging. Compared to G allele, brain FA values were significantly lower in BD patients with risk T allele in left frontal region (*P* = 0.001), right frontal region (*P* = 0.002), left parietal region (*P* = 0.001), left occipital region (*P* = 0.001), right occipital region (*P* < 0.001), and left cingulate gyrus (*P* = 0.001). Further elucidation of the interactions between different glutamate genes and their relationships with such structural, functional brain substrates will enhance our understanding of the link between dysregulated glutamatergic neurotransmission and neuroimaging endophenotypes in BD.

## 1. Introduction

Glutamate (Glu) is an excitatory neurotransmitter that is involved in important neural processes such as synaptic plasticity, neuronal development, and toxicity [1, 2]. Several studies have suggested that the abnormalities in glutamatergic function and signaling pathways through the *N*-methyl-d-aspartate (NMDA) receptors are involved in the pathophysiology of bipolar disorder (BD), a debilitating psychiatric illness characterized by alternating and often recurring episodes of mania or hypomania and depression [1, 3–6]. It was previously thought that mood stabilisers such as lithium and valproate exert their neuroprotective effects through reducing NMDA receptor-induced excitotoxicity [4–6]. Within the glutamatergic receptor, the NR2B subunit is a critical structural and functional component of the NMDA receptor. Encoded by the GRIN2B gene, which is located at 12p12 and 419 kb in size, this subunit is expressed in the cortical and medial temporal parts of the brain, striatum, and olfactory bulb [7, 8]. Earlier studies have explored the relationship between GRIN2B gene and BD [9–12]. A genetic study of Italian patients with BD found linkage to marker D12S364 at locus 12p12 within the GRIN2B gene [9]. Another study of 440 single-nucleotide polymorphisms (SNPs) from 64 candidate genes among Ashkenazi Jewish case-parent trios with bipolar I disorder noted the aforementioned association of GRIN2B with BD [10], and this was confirmed by a follow up study [11]. Genetic association studies have shown significant association between the 3′UTR region of GRIN2B and BD with psychotic symptoms [13] and number of hospitalization due to mania [14]. Recently, a positive association between GRIN2B gene and BD was also reported in Han Chinese patients with BD [12].

Understanding the impact of specific glutamatergic pathways on brain substrates in BD are important for several reasons. First, it can determine particular brain regions associated with and affected by the glutamatergic genetic signals. Second, multiplatform approaches such as genetic-imaging paradigms can clarify and highlight pathophysiological mechanisms underlying BD [15]. Third, this can subsequently foster targeted multimodality investigations involving structural, functional, and chemical neuroimaging tools. Fourth, there is also suggestion that glutamatergic genes including GRIN2B are involved in oligodendrocyte survival through common stress related signaling pathways [16]. Furthermore, previous diffusion tensor imaging studies had implicated abnormalities in brain white matter regions including frontal, parietal brain regions and cingulum in BD [17].

In the context of scant extant studies examining the impact of glutamatergic genetic signals on brain structural abnormalities in BD, we aimed to investigate the relationship of GRIN2B gene and brain white matter (WM) changes in patients with BD using diffusion tensor imaging. Based on extant neuroimaging data, we hypothesized that GRIN2B risk allele is associated with brain cortical white matter abnormalities involving reductions of white matter integrity in the frontal, parietal and temporal, and occipital regions and cingulate gyrus in BD.

## 2. Method

### 2.1. Participants

All subjects gave written informed consent to participate in the study after a detailed explanation of the study procedures. Fourteen patients suffering from BD were recruited from the Institute of Mental Health, Singapore. All diagnoses were made by a psychiatrist (K.S.) using information obtained from the existing medical records, clinical history, mental status examination, interviews with the patients, and their significant spouses or relatives as well as the administration of the Structured Clinical Interview for DSM-IV disorders-Patient Version (SCID-I/P) [18]. Participants with a history of significant neurological illness such as seizure disorder, head trauma, and cerebrovascular accidents were excluded. Furthermore, no subject met DSM-IV criteria for alcohol or other substance abuse in the preceding 3 months. The patients were maintained on a stable dose of antipsychotic medication for at least two weeks prior to the recruitment and did not have their medication withdrawn for the purpose of the study. Another twenty two age- and gender-matched healthy controls (HC) were screened using the Structured Clinical Interview for DSM-IV disorders—Nonpatient Version (SCID-I/NP)—[19] and deemed not to suffer from any Axis 1 psychiatric disorder and had no history of any major neurological, medical illnesses, substance abuse or psychotropic medication use. They were recruited from the staff population at the hospital as well as from the community by advertisements. This study was approved by the Institutional Review Boards of the Institute of Mental Health, Singapore, as well as the National Neuroscience Institute, Singapore.

### 2.2. Genotyping Procedure

PCR was performed according to Ohtsuki et al. [20] with slight modifications. Isolated genomic DNA was amplified in 25 *μ*L amplification mixture: 2 ng genomic DNA, 0.2 *μ*M of each primer, 0.5 mM of dNTPs, 0.625 U GoTaq DNA polymerase (Promega, USA), 5 *μ*L GoTaq PCR buffer, and sterile milliQ water. The cycling conditions were initial denaturation at 95 degree celsius for 2 min followed by 40 cycles with a profile of 95 degree celsius for 1 min, 59 degree celsius for 1 min, 72 degree celsius for 1 min, and a final extension at 72 degree celsius for 5 min. Amplicons (rs890G/T) were separated by electrophoresis on 1.7% agarose gel, excised, purified (Qiagen Gel Extraction Kit) and sequenced.

### 2.3. Brain Imaging Acquisition

Brain imaging was performed using a 3-Tesla whole body scanner (Philips Achieva, Philips Medical System, Eindhoven, The Netherlands) with a SENSE head coil at the National Neuroscience Institute, Singapore. High-resolution T1-weighted Magnetization Prepared Rapid Gradient Recalled Echo (MPRAGE) was required (TR = 7.2 s; TE = 3.3 ms; flip angle = 8°). Each T1-weighted volume consisted of 180 axial slices of 0.9 mm thickness with no gap (field of view, 230 mm × 230 mm; acquisition matrix, 256 × 256 pixels). For DTI, single-shot echo-planar diffusion tensor images were obtained (TR = 3725 ms; TE = 56 ms; flip angle = 90°, *b*  = 800 s/mm²) with 15 different nonparallel directions (*b*  = 800 sec/mm^2^) and the baseline image without diffusion weighting (*b*  = 0 sec/mm^2^). The acquisition matrix was 112 × 109 pixels with a field of view of 230 mm × 230 mm, which was zero-filled to 256 × 256 pixels. A total of 42 axial slices of 3.0 mm thickness were acquired parallel to anterior-posterior commissure line. The T1-weighted and DTI data were sequentially acquired in a single session scan time without position change. Stability of a high signal to noise ratio was assured through a regular automated quality control procedure.

### 2.4. Image Processing

The structural MRI images were converted from the scanned images into the Analyze format, which were further processed using the Free Surfer software package (Athinoula A. Martinos Center for Biomedical Imaging, Massachusetts General Hospital, Harvard University, http://surfer.nmr.mgh.harvard.edu/). The software reformats each brain volume image into a volume image with 1 mm^3^ isovoxels, from which relevant brain structures can be delineated [21–26]. This automated method has been shown to be statistically indistinguishable from manual raters and reduces random errors, rater error, and intersubject variability typical of manual techniques [24]. Fractional Anisotropy (FA) maps were acquired from the DTI images from the software DTI Studio [27] and were then coregistered automatically to the MP-RAGE images using a mutual information cost function and a 12 parameter affine transformation. Eddy current correction was performed prior to registration. As the DTI images are co-registered to the subjects' structural images, FA images were also automatically delineated for the separate brain structures using the same delineation parameters in the structural images and FA parameters of relevant brain structures were obtained.

The test-retest (intrarater) reliability of the measurement technique of the cortical FAs were assessed by repeated measurement of eight randomly selected subjects (4 from controls and 4 from patients) over a minimum interval of 2 weeks. On the basis of a two-factor random effects model for intraclass correlation coefficient calculation, alpha values were all greater than 0.90 for the all the cortical FA indices. Inter-rater reliability evaluation performed on a separate subset of eight subjects (4 from controls and 4 from patients) revealed alpha values of greater than 0.90 for the cortical FA indices.

### 2.5. Statistical Analyses

Demographic variables between BD and HC were compared using two sample student *t*-test and chi-square test for continuous and categorical variables, respectively. For quality control, the samples were in Hardy-Weinberg equilibrium (HWE *P* ≥ 0.05). The HWE *P*-value was obtained using the Haploview v4.2 [28], and the rests of the statistical analyses were performed using PASW 18. The genotype effect, diagnosis effect, and genotype-diagnosis interactions were further analyzed using the two-way analysis of covariance (ANCOVA) to control for covariates such as age, gender, education, handedness, and intracranial volume. Post hoc tests were performed for white matter regions with significant genotype-diagnosis interactions within HC and BD patient groups. The significance level for statistical tests was set at at two tailed *P* < 0.005.

## 3. Results

### 3.1. Sociodemographic and Clinical Characteristics

In the whole sample, there was no significant difference between BD and HC groups in age and gender. Significant difference between the groups was only found in years of education, whereby the BD group had less years of education compared to the HC group. In the BD group, the mean age of onset of the illness was 32.3 (SD 13.5) years. Overall, the mean duration of illness in BD patients was 4.07 years (SD 5.62) and the duration of untreated illness was 0.25 years (SD 0.34) (Table 1).

### 3.2. The Effect of GRIN2B Gene on White Matter Integrity in Bipolar Disorder

Overall, the T allele frequency for the GRIN2B risk variant amongst patients in the present study was 85.7%. The genotype frequencies of the GRIN2B risk variant are shown in Table 2. There were significant effects of diagnosis by genotype effect interactions observed in the bilateral frontal region (left: *F*_1,32_ = 25.5, *P* < 0.001; right: *F*_1,32_ = 18.7, *P* < 0.001), left parietal region (*F*_1,32_ = 15.8, *P* < 0.001), bilateral occipital region (left: *F*_1,32_ = 10.8, *P* = 0.002; right: *F*_1,32_ = 28.1, *P* < 0.001), and left cingulate gyrus (left: *F*_1,32_ = 18.6, *P* < 0.001). These interactions remained significant after controlling for covariates (left frontal region: adjusted *F*_1,30_ = 22.4, *P* < 0.001; right frontal region: adjusted *F*_1,30_ = 17.4, *P* < 0.001; left parietal lobe: adjusted *F*_1,30_ = 13.0, *P* = 0.001; left occipital region: adjusted *F*_1,30_ = 8.93, *P* = 0.006; right occipital region: adjusted *F*_1,30_ = 24.8, *P* < 0.001; left cingulate gyrus: adjusted *F*_1,30_ = 20.9, *P* < 0.001). (Table 3).

**Figure 1:**
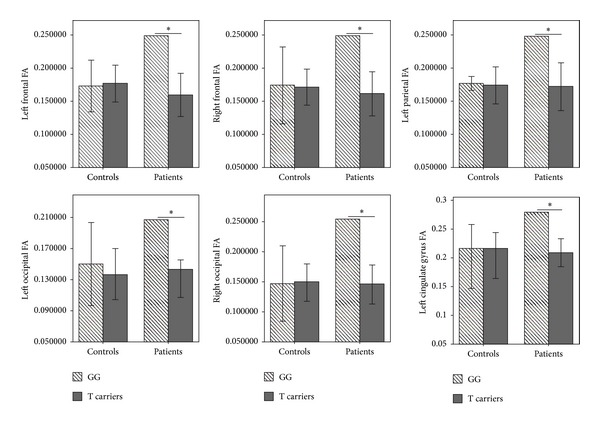
The association between GRIN2B rs890G/T genotypes and the brain white matter regions (T-bar: SD; *P < 0.005).

As the diagnosis-genotype interactions were found to be significant for bilateral frontal, bilateral occipital, left parietal regions and left cingulate gyrus, we analyzed the genotype effects on these brain regions within patient and control groups (Figure 1). There was no significant difference within the HC group; however, brain FA values were significantly lower in BD patients with risk T genotypes compared to those with G/G genotype (left frontal region: *F*_1,10_ = 24.05, *P* = 0.001; right frontal region: *F*_1,10_ = 17.85, *P* = 0.002; left parietal region: *F*_1,10_ = 9.29, *P* = 0.001; left occipital region: *F*_1,10_ = 22.19, *P* = 0.001; right occipital region: *F*_1,10_ = 33.05; *P* < 0.001; left cingulate gyrus: *F*_1,10_ = 21.50, *P* = 0.001).

## 4. Discussion

To the best of our knowledge, this is the first DTI study investigating the interrelationship between the GRIN2B risk gene variant and brain white matter abnormalities in patients with BD. We found specific significant associations between GRIN2B rs890 risk allele and brain FA reductions involving bilateral frontal regions, left parietal region, bilateral occipital regions, and left cingulate gyrus within BD patients but not in healthy controls suggesting disorder specific genetic effect on brain white matter.

Our findings are consistent with those from previous neuroimaging studies which found widespread brain white matter abnormalities in BD involving the cortical regions such as frontal, parietal, and occipital regions, as well as altered association and projection fibers although not in the context of imaging-genetic examination [17, 29–36]. A neurobiological model of affective disorders includes cortical and subcortical neural systems and can be divided into two neural networks [37]. The first is the ventral limbic network which comprises the amygdala, insula, orbitofrontal cortex, and the striatum and is responsible for identifying emotional valence of a stimulus and production of automatic affective states. The second is the dorsal cognitive network which includes the frontal cortices, anterior and posterior cingulate cortices, precuneus, and cuneus, which is involved in attention, executive and cognitive functioning [37–41]. Earlier data suggest that BD is associated with decreased activity in the dorsal network and hyperactivity in the ventral limbic network [37, 41], which can manifest as impaired performance on cognitive tasks, attention, and working memory deficits, dysregulation of mood, and abnormal emotional processing [39, 40]. Our current findings indicate GRIN2B risk allele associated reductions of white mater integrity in brain cortical regions within the dorsal network. Furthermore, the cingulate region has been hypothesized to facilitate the communication between the dorsal and the ventral systems and contribute to the regulation and integration of mood, cognitive, somatic, and autonomic responses [37]. The cingulate cortex has connections with the ventral network anatomy such as the limbic structures and facilitates top-down process of voluntary suppression/inhibition of an immediate response towards external stimuli [35, 42–45]. Disruption of white mater integrity in the cingulate gyrus may underlie increased biases towards negative and emotional stimuli or faces and diminished prefrontal modulation of affect exhibited in BD patients [37, 39].

Our study found an association between GRIN2B risk allele and lower FA in the parietal and occipital regions in BD. This is consistent with earlier DTI findings, although limited, of white matter abnormalities in the parietal and occipital regions in BD [34, 36, 46] as well as functional neuroimaging studies which have suggested abnormalities in similar parietal and occipital regions in BD [42, 43, 47]. Malhi et al. [42] performed a functional MRI (fMRI) study involving 10 euthymic BD patients and 10 matched healthy controls with the subjects engaged in a modified word-based memory task designed to implicitly invoke negative, positive or neutral affect. Compared to healthy subjects, BD patients exhibited reduced activations in the left inferior parietal lobule, right posterior cingulate gyrus, bilateral anterior cingulate gyrus, thalamus, and other cortical regions when presented with words with negative affect. Likewise, when presented with words with positive affect, BD patients showed decreased activations in the bilateral frontal gyri, right anterior cingulate gyrus, left posterior cingulate gyrus, and bilateral occipital regions compared to healthy subjects. The same research group found that poor performance during a Theory of Mind (ToM) task by euthymic BD patients was associated with less cortical activations and higher activations in the anterior cingulate gyrus and bilateral occipital regions [43]. Furthermore, a structural MRI study noted reduced gray matter density in the right parietal lobule which was associated with higher interference during the Stroop color word task in remitted patients with bipolar disorder I [47].

It was slightly surprising that no significant genotype-diagnosis interaction was noted in the temporal region despite the abundance of NR2B receptors in these regions. It is unclear how treatment with mood stabilisers such as valproate and lithium may have stabilized extant dysregulated glutamatergic neurotransmission in this region. For instance, chronic exposure to lithium was found to indirectly inhibit NMDA-receptor-mediated Ca^2+^ influx and decrease NR2B phosphorylation in temporal brain region [4–6]. Valproate induces neuroprotective proteins such as heat-shock protein (HSP70) by directly targeting histone deacetylase (HDAC) inhibition in the cortical including temporal and striatal brain regions [48].

There are several limitations to the study. First, due to the small sample of subjects our findings need to be replicated with bigger sample size. Second, analyses of other brain structural measures including cortical thickness, subcortical structures, and specific white matter tracts will complement our understanding of the impact of GRIN2B on brain white matter integrity in BD. Third, we did not correlate the structural findings with neurocognitive data which would provide better insight into the cognitive impact of GRIN2B gene in BD.

In conclusion, we found that GRIN2B was associated with reductions of brain white matter integrity within the fronto-parietal-occipital cortical regions in patients with BD. Further elucidation of the interactions between different glutamate genes and their relationships with these structural, functional, and chemical brain substrates will enhance our understanding of dysregulated glutamatergic neurotransmission and its relation to neuroimaging endophenotypes in BD. This has the potential to shed light on neurobiological mechanisms that underlie BD and provide targets for future intervention.

## Figures and Tables

**Table 1:** Demographic and clinical characteristics of participants.

Characteristics	BD (*n* = 14)	HC (*n* = 22)	Test statistic	*P *
Age^a^, years	36.9 (12.2)	32.7 (12.3)	*t* = − 0.986	.331
Gender^b^				
Males Females	10 (71.4) 4 (28.6)	11 (50.0) 11 (50.0)	*χ* ^2^ = 1.616	.204
Education^a^, years	11.4 (2.3)	14.1 (2.3)	*t* = 0.662	<0.05
Age at onset^a^, years	32.3 (13.5)	—	—	—
Duration of psychiatric illness^a^, years	4.07 (5.62)	—	—	—
Duration of untreated illness^a^, years	0.25 (0.34)	—	—	—
Medication				
Lithium Valproate	7 7	—	—	—

^a^Mean (S.D.).

^b^Mean (%).

BD: patients with bipolar disorder; HC: healthy controls.

**Table 2:** Genotype frequencies of GRIN2B risk variant rs890G/T in our sample.

Locus	SNP	Chromosome position	Genotype frequency (%)						HWE *P *
			BD (*n* = 14)			HC (*n* = 22)			
			GG	GT	TT	GG	GT	TT	
GRIN2B	rs890	13715308	1 (7.1)	2 (14.3)	11 (78.6)	3 (13.6)	5 (22.7)	14 (63.6)	0.05

BD: patients with bipolar disorder; HC: healthy controls; HWE *P*: Hardy-Weinberg equilibrium *P* value.

**Table 3:** The effects of GRIN2B of rs890G/T on brain white matter regions (mean fractional anisotropy).

	HC (*n* = 22)				BD (*n* = 14)				ANOVA (unadjusted)						ANCOVA^a^ (adjusted)					
	GG (*n* = 3)		T carriers (*n* = 19)		GG (*n* = 1)		T carriers (*n* = 13)		Diagnosis effect		Genotype effect		Interactions effect		Diagnosis effect		Genotype effect		Interactions effect	
	Mean	SD	Mean	SD	Mean	SD	Mean	SD	*F*	*P*	*F*	*P*	*F*	*P*	*F*	*P*	*F*	*P*	*F*	*P*
Left occipital lobe	0.150	0.027	0.137	0.016	0.207	—	0.131	0.012	6.99	.013	21.4	<.001	10.8	.002	7.06	.012	16.2	<.001	8.93	.006
Right occipital lobe	0.147	0.031	0.149	0.016	0.254	—	0.145	0.016	24.9	<.001	26.4	<.001	28.1	<.001	25.4	<.001	20.7	<.001	24.8	<.001
Left parietal lobe	0.177	0.005	0.174	0.014	0.248	—	0.172	0.018	13.7	.001	18.2	<.001	15.8	<.001	13.7	.001	14.2	.001	13.0	.001
Right parietal lobe	0.177	0.020	0.174	0.153	0.218	—	0.175	0.021	3.78	.061	4.34	.045	3.41	.074	3.58	.068	2.53	.122	2.68	.112
Left temporal lobe	0.164	0.004	0.163	0.009	0.163	—	0.161	0.008	0.09	.764	0.11	.743	0.01	.934	0.08	.783	0.13	.719	0.00	.956
Right temporal lobe	0.157	0.005	0.160	0.012	0.156	—	0.155	0.009	0.17	.685	0.05	.832	0.09	.771	0.17	.686	0.02	.898	0.17	.682
Left frontal lobe	0.173	0.020	0.177	0.014	0.249	—	0.159	0.016	9.96	.003	21.4	<.001	25.5	<.001	9.62	.004	19.0	<.001	22.4	<.001
Right frontal lobe	0.174	0.029	0.171	0.014	0.249	—	0.161	0.017	10.9	.002	21.6	<.001	18.7	<.001	10.8	.003	20.9	<.001	17.4	<.001
Left cingulate gyrus	0.217	0.021	0.217	0.014	0.280	—	0.208	0.012	10.9	.002	18.6	<.001	18.6	<.001	12.6	.001	24.2	<.001	20.9	<.001
Right cingulate gyrus	0.200	0.017	0.196	0.017	0.250	—	0.193	0.017	5.06	.032	8.82	.006	6.47	.016	6.86	.014	13.6	.001	7.16	.012

^a^Adjusted for age, gender, education, handedness, and intracranial volume.

BD: patients with bipolar disorder; HC: healthy controls.

## References

[1] Cherlyn SYT, Woon PS, Liu JJ, Ong WY, Tsai GC, Sim K (2010). Genetic association studies of glutamate, GABA and related genes in schizophrenia and bipolar disorder: a decade of advance. *Neuroscience and Biobehavioral Reviews*.

[2] Goff DC, Coyle JT (2001). The emerging role of glutamate in the pathophysiology and treatment of schizophrenia. *The American Journal of Psychiatry*.

[3] Clinton SM, Meadow-Woodruff JH (2004). Abnormalities of the NMDA receptor and associated intracellular molecules in the thalamus in schizophrenia and bipolar disorder. *Neuropsychopharmacology*.

[4] Hashimoto R, Hough C, Nakazawa T, Yamamoto T, Chuang D (2002). Lithium protection against glutamate excitotoxicity in rat cerebral cortical neurons: involvement of NMDA receptor inhibition possibly by decreasing NR2B tyrosine phosphorylation. *Journal of Neurochemistry*.

[5] Hokin LE, Dixon JF, Los GV (1996). A novel action of lithium: stimulation of glutamate release and inositol 1,4,5 trisphosphate accumulation via activation of the N-methyl D-aspartate receptor in monkey and mouse cerebral cortex slices. *Advances in Enzyme Regulation*.

[6] Nonaka S, Hough CJ, Chuang D (1998). Chronic lithium treatment robustly protects neurons in the central nervous system against excitotoxicity by inhibiting N-methyl-D-aspartate receptor-mediated calcium influx. *Proceedings of the National Academy of Sciences of the United States of America*.

[7] Laurie DJ, Bartke I, Schoepfer R, Naujoks K, Seeburg PH (1997). Regional, developmental and interspecies expression of the four NMDAR2 subunits, examined using monoclonal antibodies. *Molecular Brain Research*.

[8] Schito AM, Pizzuti A, Di Maria E (1997). mRNA distribution in adult human brain of GRIN2B, a N-methyl-D-aspartate (NMDA) receptor subunit. *Neuroscience Letters*.

[9] Lorenzi C, Delmonte D, Pirovano A (2009). Searching susceptibility loci for bipolar disorder: a sib pair study on chromosome 12. *Neuropsychobiology*.

[10] Fallin MD, Lasseter VK, Avramopoulos D (2005). Bipolar I disorder and schizophrenia: a 440-single-nucleotide polymorphism screen of 64 candidate genes among Ashkenazi Jewish case-parent trios. *The American Journal of Human Genetics*.

[11] Avramopoulos D, Lasseter VK, Fallin MD (2007). Stage II follow-up on a linkage scan for bipolar disorder in the Ashkenazim provides suggestive evidence for chromosome 12p and the GRIN2B gene. *Genetics in Medicine*.

[12] Zhao Q, Che R, Zhang Z (2011). Positive association between GRIN2B gene and bipolar disorder in the Chinese Han Population. *Psychiatry Research*.

[13] Martucci L, Wong AHC, de Luca V (2006). N-methyl-d-aspartate receptor NR2B subunit gene GRIN2B in schizophrenia and bipolar disorder: polymorphisms and mRNA levels. *Schizophrenia Research*.

[14] Dalvie S, Horn N, Nossek C, van der Merwe L, Stein D, Ramesar R (2010). Psychosis and relapse in bipolar disorder are related to GRM3, DAOA, and GRIN2B genotype. *African Journal of Psychiatry*.

[15] Kurnianingsih YA, Kuswanto CN, McIntyre RS, Qiu A, Ho BC, Sim K (2011). Neurocognitive-genetic and neuroimaging-genetic research paradigms in schizophrenia and bipolar disorder. *Journal of Neural Transmission*.

[16] Carter CJ (2007). eIF2B and oligodendrocyte survival: where nature and nurture meet in bipolar disorder and schizophrenia?. *Schizophrenia Bulletin*.

[17] Heng S, Song AW, Sim K (2010). White matter abnormalities in bipolar disorder: insights from diffusion tensor imaging studies. *Journal of Neural Transmission*.

[18] First MB, Spitzer RL, Gibbon M, Williams JBW (1994). *Structured Clinical Interview for DSM-IV Axis I Disorders-Patient Version (SCID-P)*.

[19] First MB, Spitzer RL, Gibbon M, Williams JBW (2002). *Structured Clinical Interview for DSM-IV Axis I Disorders-Non-Patient Version (SCID-NP)*.

[20] Ohtsuki T, Sakurai K, Dou H, Toru M, Yamakawa-Kobayashi K, Arinami T (2001). Mutation analysis of the NMDAR2B (GRIN2B) gene in schizophrenia. *Molecular Psychiatry*.

[21] Dale AM, Fischl B, Sereno MI (1999). Cortical surface-based analysis: I. Segmentation and surface reconstruction. *NeuroImage*.

[22] Fischl B, Sereno MI, Dale AM (1999). Cortical surface-based analysis: II. Inflation, flattening, and a surface-based coordinate system. *NeuroImage*.

[23] Fischl B, Sereno MI, Tootell RB, Dale AM (1999). High-resolution intersubject averaging and a coordinate system for the cortical surface. *Human Brain Mapping*.

[24] Fischl B, Salat DH, Busa E (2002). Whole brain segmentation: automated labeling of neuroanatomical structures in the human brain. *Neuron*.

[25] Fischl B, Salat DH, van der Kouwe AJW (2004). Sequence-independent segmentation of magnetic resonance images. *NeuroImage*.

[26] Fischl B, van der Kouwe A, Destrieux C (2004). Automatically parcellating the human cerebral cortex. *Cerebral Cortex*.

[27] Jiang H, van Zijl PCM, Kim J, Pearlson GD, Mori S (2006). DtiStudio: resource program for diffusion tensor computation and fiber bundle tracking. *Computer Methods and Programs in Biomedicine*.

[28] Barrett JC, Fry B, Maller J, Daly MJ (2005). Haploview: analysis and visualization of LD and haplotype maps. *Bioinformatics*.

[29] Adler CM, Adams J, DelBello MP (2006). Evidence of white matter pathology in bipolar disorder adolescents experiencing their first episode of mania: a diffusion tensor imaging study. *The American Journal of Psychiatry*.

[30] Adler CM, Holland SK, Schmithorst V (2004). Abnormal frontal white matter tracts in bipolar disorder: a diffusion tensor imaging study. *Bipolar Disorders*.

[31] Bruno S, Cercignani M, Ron MA (2008). White matter abnormalities in bipolar disorder: a voxel-based diffusion tensor imaging study. *Bipolar Disorders*.

[32] Chan WY, Yang GL, Chia MY (2010). Cortical and subcortical white matter abnormalities in adults with remitted first-episode mania revealed by tract-based spatial statistics. *Bipolar Disorders*.

[33] Lin F, Weng S, Xie B, Wu G, Lei H (2011). Abnormal frontal cortex white matter connections in bipolar disorder: a DTI tractography study. *Journal of Affective Disorders*.

[34] Regenold WT, D’Agostino CA, Ramesh N, Hasnain M, Roys S, Gullapalli RP (2006). Diffusion-weighted magnetic resonance imaging of white matter in bipolar disorder: a pilot study. *Bipolar Disorders*.

[35] Vederine F, Wessa M, Leboyer M, Houenou J (2011). A meta-analysis of whole-brain diffusion tensor imaging studies in bipolar disorder. *Progress in Neuro-Psychopharmacology and Biological Psychiatry*.

[36] Versace A, Almeida JRC, Hassel S (2008). Elevated left and reduced right orbitomedial prefrontal fractional anisotropy in adults with bipolar disorder revealed by tract-based spatial statistics. *Archives of General Psychiatry*.

[37] Mayberg HS (1997). Limbic-cortical dysregulation: a proposed model of depression. *Journal of Neuropsychiatry and Clinical Neurosciences*.

[38] Phillips ML, Drevets WC, Rauch SL, Lane R (2003). Neurobiology of emotion perception I: the neural basis of normal emotion perception. *Biological Psychiatry*.

[39] Phillips ML, Drevets WC, Rauch SL, Lane R (2003). Neurobiology of emotion perception II: implications for major psychiatric disorders. *Biological Psychiatry*.

[40] Strakowski SM, DelBello MP, Adler CM (2005). The functional neuroanatomy of bipolar disorder: a review of neuroimaging findings. *Molecular Psychiatry*.

[41] Wessa M, Houenou J, Leboyer M (2009). Microstructural white matter changes in euthymic bipolar patients: a whole-brain diffusion tensor imaging study. *Bipolar Disorders*.

[42] Malhi GS, Lagopoulos J, Owen AM, Ivanovski B, Shnier R, Sachdev P (2007). Reduced activation to implicit affect induction in euthymic bipolar patients: an fMRI study. *Journal of Affective Disorders*.

[43] Malhi GS, Lagopoulos J, Das P, Moss K, Berk M, Coulston CM (2008). A functional MRI study of theory of mind in euthymic bipolar disorder patients. *Bipolar Disorders*.

[44] Pavuluri MN, Passarotti AM, Harral EM, Sweeney JA (2009). An fMRI study of the neural correlates of incidental versus directed emotion processing in pediatric bipolar disorder. *Journal of the American Academy of Child and Adolescent Psychiatry*.

[45] Wang F, Kalmar JH, He Y (2009). Functional and structural connectivity between the perigenual anterior cingulate and amygdala in bipolar disorder. *Biological Psychiatry*.

[46] Barnea-Goraly N, Chang KD, Karchemskiy A, Howe ME, Reiss AL (2009). Limbic and corpus callosum aberrations in adolescents with bipolar disorder: a tract-based spatial statistics analysis. *Biological Psychiatry*.

[47] Haldane M, Cunningham G, Androutsos C, Frangou S (2008). Structural brain correlates of response inhibition in bipolar disorder I. *Journal of Psychopharmacology*.

[48] Chuang D (2005). The antiapoptotic actions of mood stabilizers: molecular mechanisms and therapeutic potentials. *Annals of the New York Academy of Sciences*.

